# A raster-based dataset for spatio-temporal analysis of forest fires in the Amazon rainforest from 2001 to 2020

**DOI:** 10.12688/f1000research.164537.2

**Published:** 2026-01-29

**Authors:** Mateen Mahmood, Paula Moraga

**Affiliations:** 1 Computer, Electrical and Mathematical Sciences and Engineering Division, King Abdullah University of Science and Technology (KAUST), Thuwal, Makkah Province, 23955-6900, Saudi Arabia

**Keywords:** Amazon; Fires; Burnt Area; Land Cover; Elevation; Precipitation; Humidity; Temperature

## Abstract

Forest fires are becoming increasingly common worldwide, posing a threat to the environment, economy, and society. Spatiotemporal analysis of forest fires is important to understand their characteristics and causes and to inform decision-making. This type of analysis requires the availability of a number of factors that contribute to fire occurrence, such as land use, environment, climate, and human activities, at high spatial and temporal resolutions. The South American Amazon rainforest covers a large area, and acquiring a useful dataset for analysis requires extensive effort and computer-intensive processing. This study investigates potential data sources, establishes a methodology, and prepares a dataset of attributes useful for spatiotemporal fire analysis. We provide a raster-based dataset that includes fires, land use, environment, and climate factors at a spatial resolution of 500 m and monthly temporal resolution from 2001 to 2020, which facilitates the analysis of forest fires in the Amazon. Moreover, because data sources and implementation procedures are detailed, this work also encourages similar research in other parts of the world.

## Introduction

The alarming increase in the frequency and severity of forest fires around the globe has become a significant threat to forested areas worldwide. These wildfires not only threaten human lives and their properties but also continue to contribute to the reshaping of local and global ecosystems. Because of their varying spatiotemporal nature at multiple scales, they are substantially diverse in their frequency, size, intensity, and pattern. ^1^ Similarly, the source of ignition is an amalgamation of numerous aspects such as weather, climate, land use, and other causes such as lightning, volcanic eruptions, rockfalls, and combustion material. ^2^ This constant vulnerability of forests exposed to wildfires is horrifying, but when considered in the context of ecological and socio-economic consequences, it poses a major challenge to fire management authorities and related stakeholders. ^3^

To ensure better preparedness and deploy improved preventive measures, the spatio-temporal relations between the probable causes of wildfires and the characteristics of those fire incidents must be analyzed. Such analysis will not only assist with mitigation but may also aid in the prediction and forecasting of future events by better understanding the underlying events propagating fire occurrences. ^4^ Such in-depth spatio-temporal statistical investigations of these complex interactions require the collection of all available associated attributes, combined from heterogeneous sources (with varying extents, spatial scales, temporal resolutions, file formats, etc.) into a processed unified structure available in the form of common specifications.

The South American Amazon is one of the largest rainforests in the world ^5^ and hosts thousands of wildfires annually. ^6^ Despite numerous studies related to spatio-temporal statistical analysis of forest fires in many regions of the world, ^2, 4, 7– 9^ there remains a notable scarcity of basin-wide, multivariate longitudinal studies for the entire Amazon region. While some research has addressed specific drivers of ignition, existing Amazon-specific studies tend to be limited to sub-regions or specific administrative boundaries. ^3, 10^ For a study area of this size, data collection is a time-intensive task, with exhaustive pre-processing requiring cumbersome setups. Hence, the development of an Amazon-wide database that includes all available attributes related to fires, integrated into a common format, is required.

**Figure 1.**
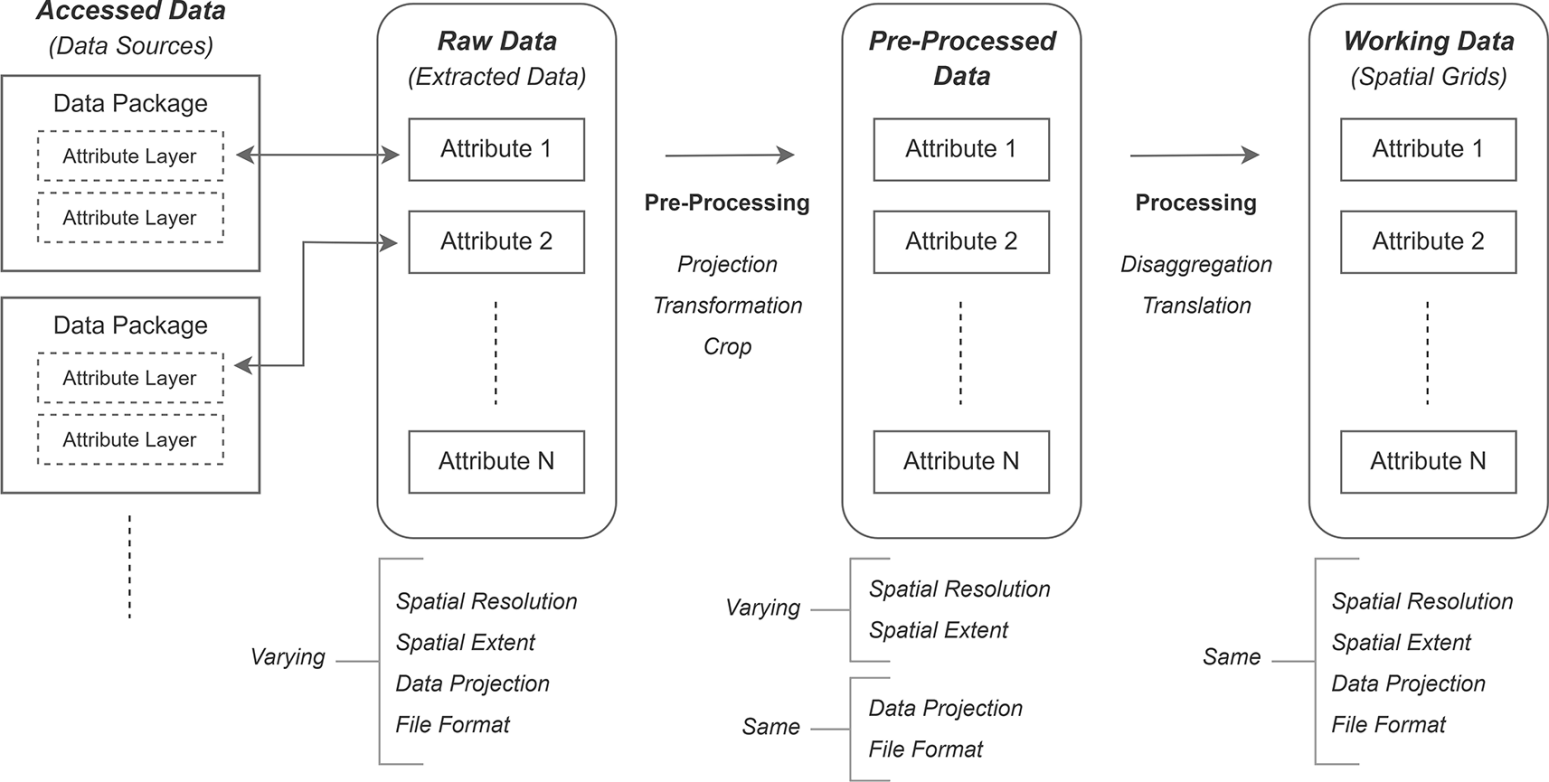
Schematic overview of the data processing process.

The aim of this work is to provide a scientific community with a dataset related to spatiotemporal forest fire analysis for the Amazon region. The dataset includes historical data of 20 years (2001-2020) in a monthly temporal resolution for the complete extent of the Amazon region at a spatial scale of 500 m. Because the study area of the entire Amazon rainforest is large, the raw data sources must be at a global or regional level (in South America). Otherwise, data for the same attribute are expected to be gathered from multiple local-level sources, raising concerns regarding data integrity. Global- and regional-level satellite-based raster products were acquired and further clipped for the South American region to compute three types of data: *(a) raw data*, *(b) pre-processed data* and *(c) working data.* A schematic overview of this study is presented in Figure 1. *Raw data* refer to data file(s) extracted from the accessed data packages (i.e., data layer of the subject attribute, taken out from the data package containing various other attribute layers as well). The extracted attribute layers have varying spatial resolutions, dissimilar spatial extents, different spatial projections, and inconsistent file formats. Raw data are pre-processed to acquire *pre-processed data*, with the attribute layers in a consistent file format and with the same projection system. Finally, all attribute layers are processed to obtain *working data*, with the data extent confined to the Amazon region and with fixed spatial resolution, such that each raster cell of an attribute layer aligns exactly over the raster cell of the other attribute layer.

This manuscript presents the complete process of data collection for raster-based attributes of forest fires in the Amazon rainforest, along with a description of the methodological baseline and details of the implementation process. The availability of such a ready-made dataset with a detailed methodology of data collection and computer-intensive preprocessing procedures will be useful to many researchers working in the domain of forest fire analysis. For example, this dataset has been used to map the geographic and temporal distributions of burned areas and risk factors in the Amazon from 2001 to 2020 using an ensemble approach that harnesses a range of machine learning algorithms. ^11^ Furthermore, this dataset provides encouragement for developing similar datasets tailored to varying study regions, spatial resolutions, and research domains. ^12^

## Methods

**Figure 2.**
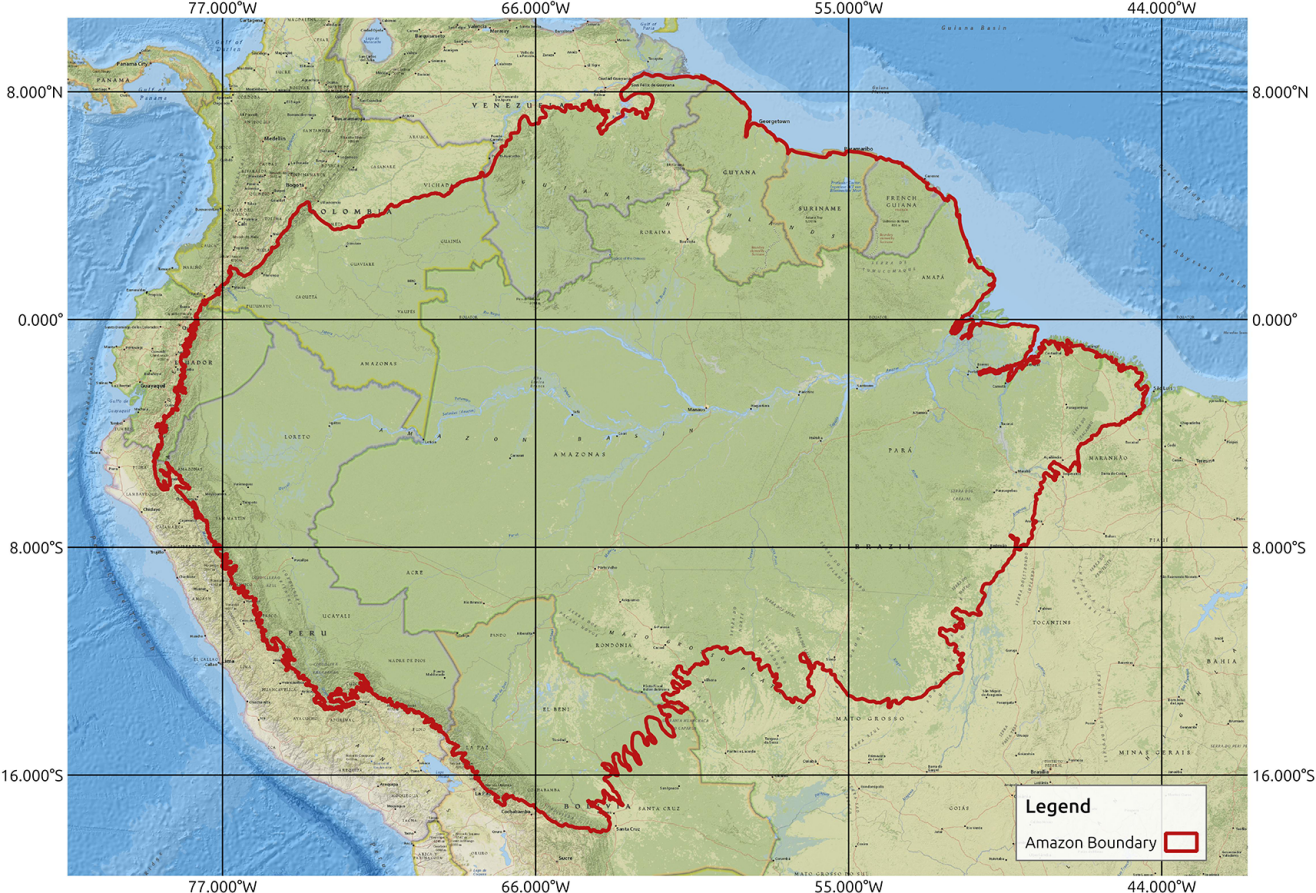
Study area of Amazon rainforest. Amazon boundary obtained from. ^20^

The Amazon rainforest has an area of over 5.2 million square kilometers, covers approximately one-third of South America, and extends into eight countries. ^5^ Within this region, data management authorities in each country generally focus on their own regions. To create a database for the entire extent of the Amazon rainforest and to ensure that all relevant areas of potential importance are included in the study area, we defined the study area for this work as the entire Amazon basin, as shown in Figure 2. The extent of the study area can be defined as -79.43629, -18.00816: -44.49108, 8.66346 with the coordinate reference system EPSG:4326 - World Geodetic System (WGS) 84 - Geographic. For spatiotemporal modeling, the selection of the data period needs to have a considerable temporal range as well as data availability for the chosen period. A review of the literature related to spatiotemporal modeling of forest fires, as summarized in Table 1, indicates that a period of 5-30 years with monthly or yearly frequency is used for the temporal characterization of forest fires. Keeping in view what is available for the Amazon Rainforest (for the whole region), we decided to proceed with a data period of 20 years from 2001 to 2020, with a monthly frequency as the temporal resolution. The spatial resolution was finalized as 500 m for the final *spatial grid.* This is based not only on the available data for the Amazon Rainforest but also on the computational complexity involved in a study area of approximately 5 million square kilometers.

**Table 1.** Summary of study characteristics from previous works related to forest fire analysis.

Reference	Study region	Study area	Data period	Temporal resolution
A ^4^	Southern France	40,000 sq.km	1995-2018	Monthly
B ^21^	Autazes, Brazil	7,632 sq.km	1985-2015	Monthly
C ^22^	South Korea	99,720 sq.km	1980-2000	Annual
D ^2, 7, 23^	Catalonia, Spain	30,000 sq.km	2004-2008	Multi-Year
E ^8^	Castellon, Spain	6,632 sq.km	2001-2006	Multi-Year
F ^24^	Islamabad, Pakistan	158 sq.km	2005-2018	Multi-Month
G ^25^	California and Nevada, USA	120,000 sq.km	1984-2006	Multi-Month

In addition to the study design involving spatial resolution, temporal frequency, and spatial data extent, another equally important aspect is the selection of covariates. These variables can be broadly categorized as attributes related to land use, climate, the environment, topography, and human activities. Land use and land cover (LULC) variables are highly related to forest fires, as the type of land surface not only determines fire ignition but also its propagation. Climatic variables, such as humidity, precipitation, wind speed, and temperature, also influence the occurrence of forest fires. Topographic variables such as elevation, slope, and aspect are also of core importance as they regulate how quickly a fire will move up or down the hills. Finally, human activities also play a critical role in the initiation of forest fires. Hence, variables such as population density, buildings, and the urban-forest interface are of high significance. Table 2 summarizes the list of potential forest fire analysis attributes discussed in the literature.

**Table 2.** Summary of study attributes from previous works related to forest fire analysis.

Reference	Description of attributes
F ^3, 8, 9, 26, 27^	Land Use Effects/Vegetation Type/Deforestation/Forest Type/Land Cover
G ^4, 9, 26^	Population Density/Housing Density/Buildings
H ^3, 4, 26, 27^	Elevation, Slope and Aspect
I ^9^	Humidity
J ^9^	Wind Speed
K ^4, 9, 26, 27^	Temperature
L ^4, 9^	Precipitation
M ^8^	Isothermality
N ^4^	Protected Zones
O ^3, 8, 9, 26^	Road Density, Distance to Road
P ^3^	Maximum Cumulative Water Deficit
Q ^3, 8^	Soil Type/Soil Texture/Soil Permeability

### Data collection

From the list of attributes identified from the literature as potentially related to forest-fire analysis ( Table 2), not all of them are available for the entire Amazon Rainforest, let alone for the study period 2001-2020. Specifically, variables such as protected zones, isothermality, and maximum cumulative water deficit were only available for certain regions and for a particular time period. Similarly, elevation-related attributes were only available for certain years between the period 2001-2020. In this study, attributes that were available for the complete Amazon region and for the selected time period of 2001-2020, are identified and further acquired, as detailed in Table 3, with *Date of Access: 01 May 2022.* This section details the complete data-acquisition process related to each collected attribute.

**Table 3.** Summary of collected attributes related to forest fire analysis, with original temporal resolution of monthly frequency (except Land Cover which is Annual, and Elevation which is One time). These attributes were pre-processed to acquire *working data* at 500 meters and monthly resolution, for the period of 2001 to 2020.

S#	Variable name	Description	Spatial resolution	Source
1.	Burnt Area	Classes (Burnt, Not Burnt, Water)	500 meters	MODIS ^28^
2.	Land Cover (Annual)	11 Classes of Land Cover	5,600 meters	MODIS ^29^
3.	Precipitation	Average rate of precipitation	10,000 meters	GES-DISC ^30^
4.	Soil Moisture	Model-calculated	37,000 meters	CPC ^31^
5.	Elevation (One-time)	Based on Digital Elevation Model	1,000 meters	EarthEnv ^32^
6.	Land Surface Temperature	Daytime observations	5,000 meters	MODIS ^33^
7.	Specific Humidity	Model-calculated	1,000 meters	GES DISC ^34^
8.	Evapotranspiration (ET)	Model-calculated	1,000 meters	GES DISC ^34^
9.	Near Surface Wind Speed	Model-calculated	1,000 meters	GES DISC ^34^
10.	Near Surface Air Temperature	Model-calculated	1,000 meters	GES DISC ^34^

***Burnt Area (BA)***

The data product acquired was MODIS/Terra+Aqua Direct Broadcast Burned Area Monthly L3 Global 500 m SIN Grid V006 MCD64A1 Version 6.1, which is a gridded burnt area product at a resolution of 500 m, available in Hierarchical Data Format (HDF) format. The product provides the date of burn (in the form of the day of the year) for individual cells with additional classes, such as unburnt, missing data, and water. The data product is available for the period 2000 to the present (2022), with global spatial coverage in the form of regional subsets. The layers extracted from the data source are for regions 5 and 6, which cover the Amazon area. The data layer values are in units of a day, with a valid range of data values as between 1-366 (representing the day of the year). Further details related to the product, including the quality assessment and known issues, are available at MODIS MCD64A1 ( https://lpdaac.usgs.gov/products/mcd64a1v061/ ).

As the burnt area product is available at the regional level, an additional data processing step for the burnt area product is the merging of two separate regional-level products to cover the entire region of the Amazon basin boundary. Additionally, the data were re-classified to assign a single value of 1 to all burn dates (1-366) to identify the cell with burn data as simply burnt. Hence, working data has four classes (burnt, unburnt, missing, and water) with values (1, 0, -1, and -2), respectively.

***Land Cover (LC)***

The data product acquired was MODIS/Terra+Aqua Land Cover Type Yearly L3 Global 0.05Deg CMG V006 MCD12C1 Version 6, which consists of three gridded land cover classification schemes at a resolution of 5,600 m, available in the HDF format. The three available classification schemes include *Maps of the International Geosphere-Biosphere Programme (IGBP)* providing 17 classes, *University of Maryland (UMD)* providing 16 classes, and *Leaf Area Index (LAI)* providing 11 classes. LAI classification schemes are extracted from the data product as 11 classes are sufficient for representation of different land covers in terms of Water, Urban, Forest, Grassland, etc., and additional classes available in other schemes are further subdivisions of forests and grassland types. The data product is available for the period 2000 to the present (2022) with global spatial coverage. The details of the land cover classes of the LAI scheme are provided in Table 4. The name of the layer extracted from the data source is Land Cover Type-3, with a range of data values between classes 0 and 10. Further details related to the product, including the quality assessment and known issues, are available at MODIS MCD12C1 ( https://lpdaac.usgs.gov/products/mcd12c1v006/ ).

**Table 4.** Class details of Leaf Area Index (LAI) classification scheme, from MODIS. ^29^

Class name	Value	Description
Water Bodies	0	Permanent water bodies
Grasslands	1	Dominated by herbaceous annuals (<2 m)
Shrublands	2	Shrub (1-2 m)
Broadleaf Croplands	3	Dominated by herbaceous annuals (<2 m) - cultivated with broadleaf crops
Savannas	4	From 10% to 60% tree cover (>2 m)
Evergreen Broadleaf Forests	5	Dominated by evergreen broadleaf and palmate trees (>2 m)
Deciduous Broadleaf Forests	6	Dominated by deciduous broadleaf trees (>2 m)
Evergreen Needleleaf Forests	7	Dominated by evergreen conifer trees (>2 m)
Deciduous Needleleaf Forests	8	Dominated by deciduous needleleaf (larch) tree (>2 m)
Non-Vegetated Lands	9	Non-vegetated barren (sand, rock, soil) /permanent snow and ice
Urban and Built-up Lands	10	Impervious surface area including building materials, asphalt, and vehicles
Unclassified	255	Missing inputs

***Precipitation***

The data product acquired is Integrated Multi-satellite Retrievals for GPM (Global Precipitation Measurement)-based multi-satellite precipitation product, Version 06 B, available in Hierarchical Data Format version 5 (HDF5) format. The product provides a monthly product of average precipitation rates at a 0.1 °× 0.1 ° (approximately 10,000 m at the equator) spatial resolution, estimated from numerous precipitation-relevant satellite passive microwave (PMW) sensors. The dataset is available for 2000–2021 with global spatial coverage. The values are represented in *millimeters per hour* ( *mm/hr*), with a scale factor of 1000 and missing values marked with -9999. Thus, a value of 500 indicates 500/1000 mm/h. Further details related to the product are available at the GES-DISC GPM IMERG Final Precipitation L3 ( https://disc.gsfc.nasa.gov/datasets/GPM_3IMERGM_06/summary ).

***Soil moisture***

The data product acquired is a model-calculated (not directly observed) averaged soil moisture water height equivalent, namely CPC Soil Moisture Version 2, available in the GEOTIFF format. The data are a monthly product of 0.5 °× 0.5 °(approximately 37,000 m at the equator) spatial resolution, with data available from 1948 to the present (2022). The spatial coverage of the product is 89.75N–89.75S, 0.25E–359.75E. The values are represented in *millimeters* ( *mm*), with missing values marked as -9999. Further details related to the product are available at CPC Soil Moisture ( https: //psl.noaa.gov/data/gridded/data.cpcsoil.html ).

In the preprocessing of the Soil Moisture data product, data transformation is implemented as an additional step. As the source data have a spatial offset, not aligning with the reference base map, the data are transformed to correct alignment using the Geospatial Data Abstraction Library (GDAL). ^13^

***Elevation***

The acquired data product is a global multivariate package related to terrain features, which can serve many large-scale research publications. The data product is based on a 250 m Digital Elevation Model (DEM), available in Tagged Image File Format (TIF) format, from Global Multi-Resolution Terrain Elevation Data 2010 (GMTED2010). ^14^ This data product provides many topographic variables, such as elevation, slope, aspect, northness, elasticity, roughness index, and topographic position index at different resolutions of 1, 10, 50, or 100 km, with global spatial coverage; however, our focus is only on elevation. The Elevation values are represented in *meters* ( *m*). Further details related to this product are available at ( https://www.earthenv.org/topography ).

***Land Surface Temperature (LST)***

The data product acquired was MODIS/Terra Land-Surface Temperature/Emissivity Monthly Global 0.05Deg CMG MOD11C3 Version 6, which is a monthly Land Surface Temperature & Emissivity (LST&E) value product at a spatial resolution of 0.05 ° (approximately 5,600 m), available in the HDF format. The data product provides values for both daytime and nighttime observations, along with other details related to the quality assessment. The data product is available for the period 2000 to the present (2022) with global spatial coverage. The temperature values are represented in *kelvin* ( *K*), with a scale factor of 0.02 and a range of values between 7,500 and 65,535. Thus, the LST value equal to X represents X*0.02 kelvin. Further details related to this product are available at MODIS MOD11C3 ( https://lpdaac.usgs.gov/products/mod11c3v006/ ).

***Specific humidity, Evapotranspiration (ET), wind and air temperature***

The acquired data provides a set of parameters related to land surface observations. The data is a simulation-based product of the Noah 3.6.1, model from Famine Early Warning Systems, Network (FEWS NET) Land Data Assimilation System (FLDAS). All the provided variables are available as a monthly product in a 0.10 degree spatial resolution (approximately 1,000 m at the equator) and available (as a layer) in NETCDF file format. The dataset is available for the period from 1982 to the present (2022) with global spatial coverage. The values of Specific Humidity are represented as ( *kg/kg*), using a ratio between kilogram of water (moisture) per kilogram of air; whereas Evapotranspiration, Wind and Air Temperature are measured in ( *kg/m* ^2^ *s*), ( *m/s*) and *kelvin* ( *K*), respectively. Further details related to the product are available at the GES DISC-FLDAS Noah Land Surface Model L4 ( https://disc.gsfc.nasa.gov/datasets/FLDAS_NOAH01_C_GL_M_001/summary ).

While these model-derived variables (including Soil Moisture) introduce inherent numerical uncertainties compared to direct field observations, they are incorporated to expand the suite of available environmental covariates, providing the multivariate depth necessary for robust spatiotemporal analysis across the Amazon. As the primary objective of this work is the curation and standardization of a basin-wide Amazon dataset, a formal independent uncertainty analysis remains beyond the scope of this data curation effort. By providing these products in a common, analysis-ready format, this work establishes the necessary foundation for future studies to conduct such analytical sensitivity assessments and empirical validations.

### Data processing

All of the various attributes collected in the database have different spatial resolutions, as described in Table 3. Similarly, not all variables are available at monthly resolution, as Land Cover and Elevation are annual and one-time, respectively. Moreover, all of these variables cover different spatial extents and have dissimilar spatial orientations. To obtain a dataset with all the variables at a fixed spatial extent and resolution, we constructed a spatial grid of 500m resolution covering the Amazon region and obtained the cell values for this raster following the steps described below. Similarly, we executed the process to achieve a monthly temporal resolution for all variables, with the data period from 2001 to 2020.

To achieve temporal harmonization across these differing frequencies, we adopted a *temporal expansion* framework where annual and static values are mapped consistently across the corresponding twelve monthly increments of each year. This ensures that every monthly snapshot in the 240-month time series contains a complete suite of environmental covariates, enabling dynamic analyses such as fire risk modeling or temporal trend assessment. This approach maintains temporal sensitivity by allowing dynamic climate variables to fluctuate monthly while the slower-evolving landscape attributes such as Land Cover provide a stable structural context for each year.

**Figure 3.**
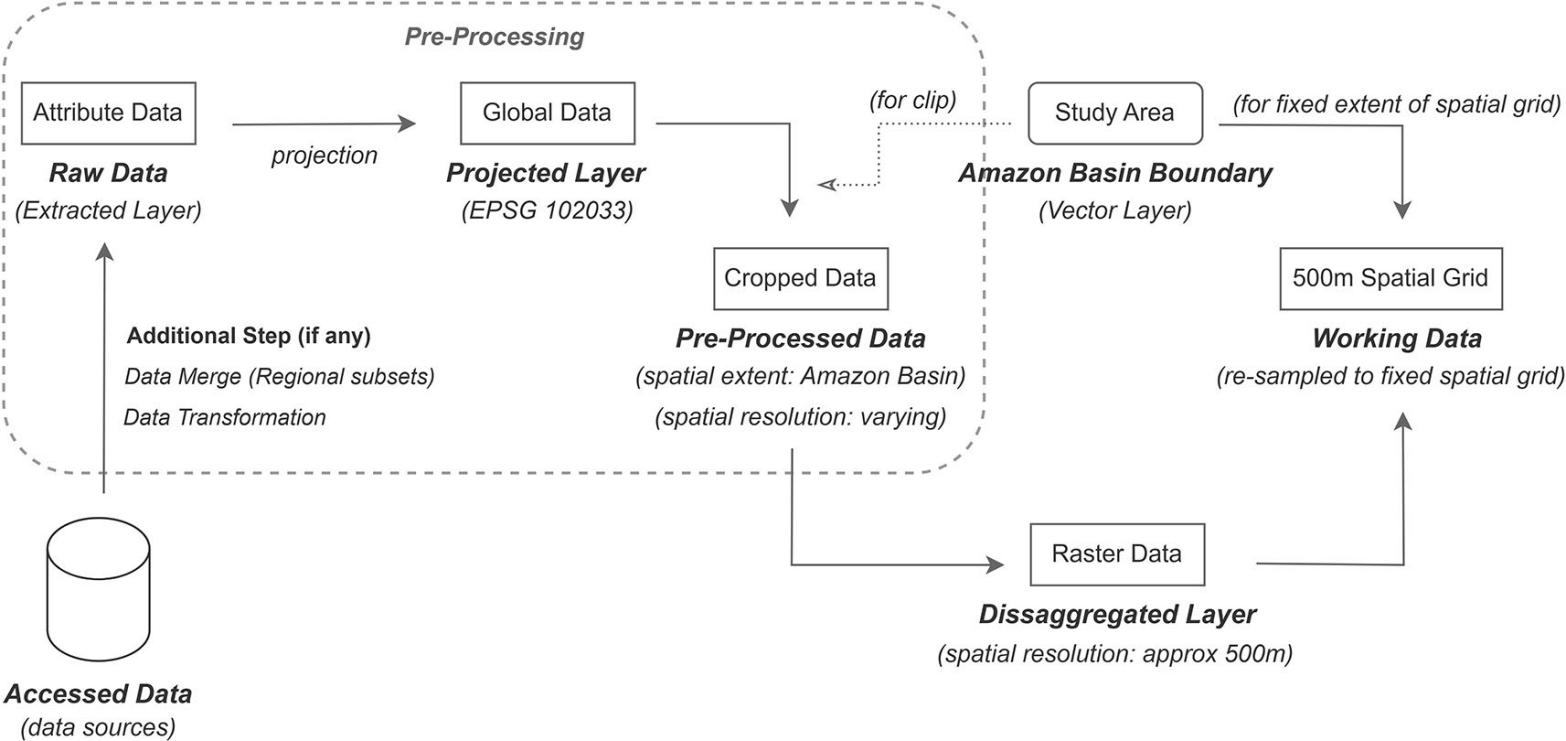
Overview of methodology for data processing.

Although the collected data packages for different attributes have heterogeneous specifications, their processing generally follows a common workflow. A methodological baseline of the processing steps is shown in Figure 3. Specifically, *Accessed Data* refers to the downloaded data package from data sources in various formats, such as HDF, HDF5, NETCDF, GEOTIFF, and TIF. Accessed data in source data formats, such as HDF, HDF5, and NETCDF, contained several layers with different attributes, and the layer related to the subject attribute was extracted from this set of layers. Accessed data with the source data formats of GEOTIFF or TIF contained only the required layer that was extracted. These extracted layers are referred to as the *raw data.*

To resolve the inherent inconsistencies in spatial orientation and resolution, we developed a standardized processing framework. First, all *raw data* layers are projected onto EPSG:102033-South America Albers Equal Area Conic, to acquire the *Projected Layer*. This equal-area coordinate reference system was specifically chosen to maintain geometric fidelity across the vast longitudinal expanse of the Amazon basin, ^15^ minimizing the distortion of area and shape that typically occurs in global projections. To correct for grid misalignments and boundary distortions caused by the differing source orientations of the datasets collected, we introduced a standardized spatial grid as a master template. By mapping all attributes onto this fixed spatial grid, we ensured that every pixel across all variables represents the exact same geographical footprint, thereby eliminating spatial offsets and ensuring seamless interoperability between datasets. 

The projected layers are at either a global or regional-level (based on the specifications of the data source), and to confine them all to the Amazon Basin boundary, these layers were further clipped using a shapefile-based (vector) Amazon Basin boundary. This clipped layer is labelled as *pre-processed data*.

Although all layers are cropped to the Amazon basin boundary, their respective cells may not exactly align with each other owing to differences in their source data extent, cell-grid orientation, and spatial resolution. To obtain layers of the same spatial extent, resolution, and orientation, we executed a rigorous two-step disaggregation and resampling procedure to transfer cell values from the *pre-processed data* to the fixed spatial grid (master template). The spatial grid covered the entire Amazon Basin boundary and had a cell resolution of 500 m. The value of each cell was transferred to this grid for each attribute, and the process was repeated for all attributes, thereby creating a separate spatial grid for each attribute.

First, layers with resolution coarser than 500 m (ranging up to 37 km) were disaggregated to approximately 500 m. The disaggregation factor varied for each attribute, based on the spatial resolution of the source data. Following this, the terra:resample function in R was used to transfer information from the attribute layers to the fixed spatial grid. To minimize spatial inaccuracy in this environmentally heterogeneous region, the resampling method was tailored to the variable type: the ‘near’ (nearest neighbor) method was employed for Land Cover, Burnt Area, Soil Moisture, Specific Humidity, Evapotranspiration, Near Surface Wind Speed, and Near Surface Air Temperature to preserve original discrete values and categorical integrity. Conversely, ‘bilinear’ interpolation was used for Land Surface Temperature and Precipitation to accurately represent the continuous spatial gradients of these atmospheric phenomena.

The resulting spatial grids corresponding to each attribute constitute the final layers available for analysis, hence called *working data*. This workflow was followed for each monthly file (i.e., 240 files over 20 years) to achieve a consistent monthly temporal resolution from 2001 to 2020. By maintaining this monthly sensitivity for dynamic climate variables while holding slower-evolving landscape variables such as Land Cover constant within each annual cycle, the dataset remains sensitive to the immediate environmental drivers for fire while providing a stable structural context. While an analytical sensitivity assessment regarding the impact of integrated variable frequencies is a valuable research direction, such an analysis is beyond the scope of this data curation work.

**Figure 4.**
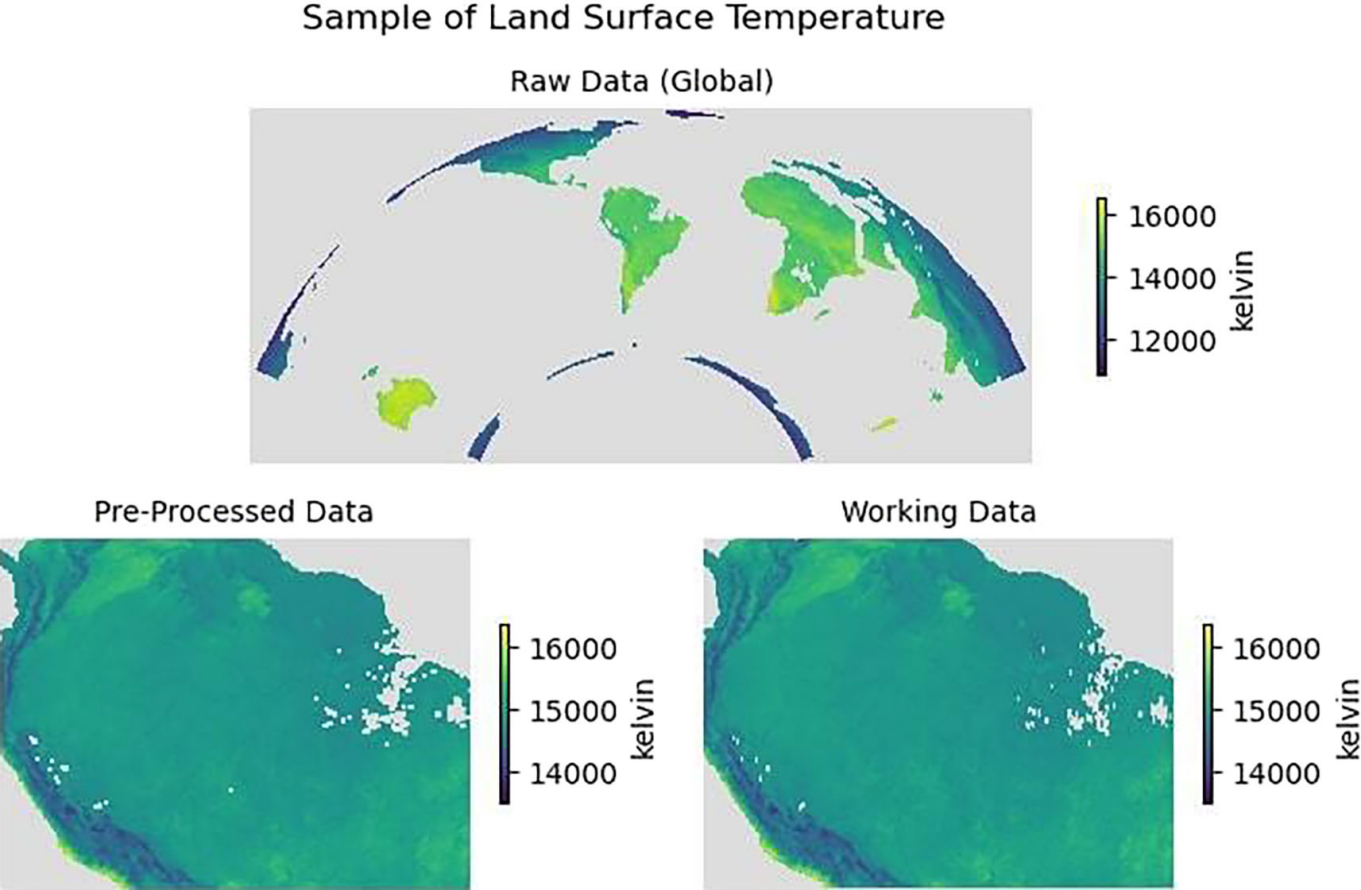
Land surface temperature for January 2020. *Top*: Raw data (Global), *Bottom*: Pre-processed data (cropped) and working data (re-sampled spatial grid).

**Figure 5.**
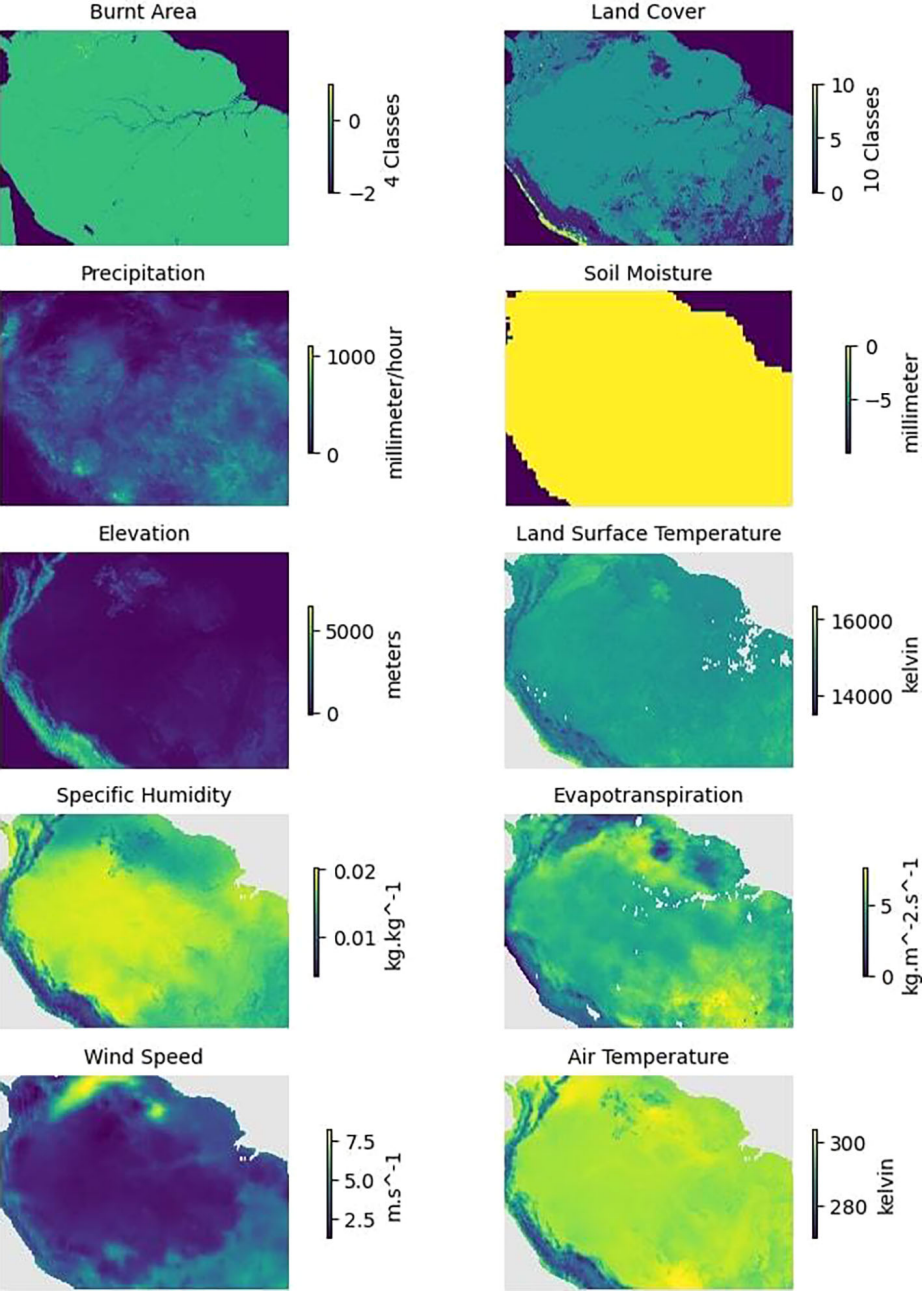
Plots of the variables related to forest fires for the region of Amazon Rainforest in January 2020.

Figure 4 illustrates an example of Land Surface Temperature in January 2020 for all three categories of *raw data*, *pre-processed data* and *working data.* Similarly, Figure 5 presents an example of a single monthly instance from January 2020 for all the variables collected.

In terms of implementation, pre-processing work was completed using GIS software, and the processing work was executed in the statistical computing software R. ^16, 17^ All data layers were managed using the SpatRaster data structure in the terra package, ^18^ ensuring a transparent, scripted workflow. This algorithmic approach serves as a systematic process log, minimizing accumulated errors across the 240 temporal layers and ensuring the reproducibility of the dataset.

### Technical validation

The raster-based dataset of covariates presented in this study is a collection of established datasets that do not include any newly created data records. This work mainly focuses on exhaustive data search and its acquisition process, followed by computer-intensive pre-processing to develop a dataset for the Amazon region. As noted in the Data Collection section, these industry-standard source datasets have undergone rigorous independent validation, as documented in their respective technical documentation; therefore, a secondary validation against field observations is beyond the scope of this curation effort. To ensure process transparency, the dataset follows standardized naming conventions.

While the primary contribution of this work lies in workflow standardization and the resolution of dataset fragmentation, this technical framework serves as the essential infrastructure required for future algorithmic advancements. By delivering a harmonized, analysis-ready foundation, this study enables high-level environmental research and predictive modeling, ^11^ that were previously hindered by spatial and temporal data incompatibility. This standardized curation ensures that the resulting *working data* is fit-for-purpose for complex spatiotemporal analyses and provides a reproducible baseline for the wider research community.

## License

The raster-based dataset of covariates presented in this study was published under a Creative Commons Attribution 4.0, International (CC BY 4.0) License ( https://creativecommons.org/licenses/by/4.0/), which permits use, sharing, adaptation, distribution, and reproduction in any medium or format, as long as appropriate credit is given to the authors and the source, a link to the license is provided, and it is indicated if changes were made.

## Data Availability

The dataset with all the collected variables related to forest fires is available at the Zenodo repository titled ‘Raster-based dataset for spatio-temporal analysis of forest fires in the Amazon rainforest from 2001 to 2020’ ( https://doi.org/10.5281/ zenodo.7215402 ). ^35^ The dataset comprises three folders for each of the ten variables, referring to the data categories of *Raw Data*, *Pre-Processed Data* and *Working Data* with names *01. Raw Data*, *02. Pre-Processed Data* and *03. Working Data*, respectively. An additional *Read Me* document includes details regarding the coordinate system, data extent, and data sources. All files were in GEOTIFF format, which can be accessed using the statistical software R ^16^ or any of the GIS software, such as Quantum GIS - QGIS (opensource) ( https://www.qgis.org/en/site/ ), GRASS GIS (opensource) ( https://grass.osgeo.org/ ), or ArcGIS (proprietary) ( https://www.arcgis.com/index.html ). In the case of Land Cover, which is annual-based data, the filename includes the variable short name (Landcover), the respective data category (raw for raw data, preproc for pre-processed data, or working for working data), and the year (2001–2020):

[Landcover]_[data_category]_[year].tif In the case of elevation, which is only one-time data, the filename includes the variable’s short name and the respective data category:

[Elevation]_[data_category].tif For all other variables, the filename includes the variable short name, the respective data category, and the year and month:

[variable_short_name]_[data_category]_[year]_[month].tif To load and visualize the data in R, .tif files of any of the three categories can be loaded as a raster by using the raster ^19^ or terra packages. The plot function of terra can be used to visualize the raster as follows:
r <- terra::rast(’<filepath/filename.tif>’) plot(r) Similarly, to visualize the data in Quantum GIS (QGIS), .tif file can be loaded to select the raster option in the Data Source Manager:
[Data Source Manager > Raster > (filepath)]
