# Supplementary material for: A raster-based dataset for spatio-temporal analysis of forest fires in the Amazon rainforest from 2001 to 2020
Source: F1000Res. 2026 Jan 29;14:916. Originally published 2025 Sep 12. [Version 2] doi: 10.12688/f1000research.164537.2 (PMC13231137; doi:10.12688/f1000research.164537.2)
Supplement: Peer review history

# Reviewer report: Reviewer response for version 2

**DOI:** 10.5256/f1000research.195424.r482336

**Published:** 2026-06-02

**Authors:** Suresh Babu KV Babu

**Affiliations:** 1 University of Cyprus, Nicosia, Cyprus

This work presents a large-scale, harmonized, analysis-ready dataset designed for forest fire research in the Amazon basin. It integrates 20 years of monthly environmental and fire-related variables at a spatial resolution of 500 meters across the entire Amazon region. The dataset fills a major gap in basin-wide wildfire research by giving researchers a standardized and reproducible way to do multivariate spatiotemporal analysis.

 By converting heterogeneous raw data into a consistent format, including projection, extent, and resolution, the study significantly facilitates researchers’ investigations into wildfire dynamics in the Amazon. Additionally, it offers a valuable methodological framework for creating similar datasets in other regions or for various environmental applications.

Are sufficient details of methods and materials provided to allow replication by others?

Yes

Is the rationale for creating the dataset(s) clearly described?

Yes

Are the datasets clearly presented in a useable and accessible format?

Yes

Are the protocols appropriate and is the work technically sound?

Yes

Reviewer Expertise:

Wildfire risk modeling, fire danger prediction, Burned area, Fire forecasting, Fire detection, Burned area mapping etc.

I confirm that I have read this submission and believe that I have an appropriate level of expertise to confirm that it is of an acceptable scientific standard.

# Reviewer report: Reviewer response for version 1

**DOI:** 10.5256/f1000research.181063.r422841

**Published:** 2025-11-08

**Authors:** Mahlatse Kganyago

**Affiliations:** 1 University of Johannesburg, Johannesburg, South Africa

**A raster-based dataset for spatio-temporal analysis of forest fires in the Amazon rainforest from 2001 to 2020**

 The authors present a curated dataset of the amazon basin including various landscape and climate variables necessary to model long-term trends of forest fires. The paper is generally well-written and methods can be replicated in other regions.

**Abstract**

 “and monthly resolution” – I suggest that authors add temporal after monthly.

**Introduction**

 “The South American Amazon is one of the largest…” – should start as new paragraph since it diverts from the idea communicated in this paragraph.

 “similar studies do not exist for the Amazon region” – Please confirm through a comprehensive literature search. I doubt this is entirely accurate.

 “this dataset encourages the creation of similar datasets” – please rephrase to improve clarity.

**Methods**

 “[-79.43629, -18.00816: -44.49108, 8.66346]” – Should not be in brackets.

 “MCD64A1” , “MCD12C1”, “MOD11C3” – please provide name of the product in addition to its ID or Acronymn.

  “…acquired is (Integrated Multi-satellite Retrievals for GPM (Global Precipitation Measurement (GPM)…” – Should not be in brackets.

 [89.75N–89.75S, 0.25E–359.75E] - Should not be in brackets.

 “…as –  -9999.” – not clear, please consider removing the dash.

 “Noah 3.6.1, model…” – the comma here creates fragmentation.

 “…and are termed Working Data” – The term has been used above, but only defined here. Please consider defining a term when they are first mentioned.

 “file (240 files over 20 years)” – please insert “i.e.,” in brackets.

**Data availability**

 There is space on the data link which breaks the hyperlink. Please correct.

https://doi.org/10.5281/ zenodo.7215402

Are sufficient details of methods and materials provided to allow replication by others?

Yes

Is the rationale for creating the dataset(s) clearly described?

Yes

Are the datasets clearly presented in a useable and accessible format?

Yes

Are the protocols appropriate and is the work technically sound?

Yes

Reviewer Expertise:

Remote sensing of vegetation

I confirm that I have read this submission and believe that I have an appropriate level of expertise to confirm that it is of an acceptable scientific standard, however I have significant reservations, as outlined above.

# Response

**Published:** 2026-01-18

**Authors:** Paula Moraga

**Affiliations:** King Abdullah University of Science and Technology Computer Electrical and Mathematical Science and Engineering Division, Thuwal, Makkah Province, Saudi Arabia

Thank you for your helpful and insightful comments. Please find our responses below.

 “and monthly resolution” – I suggest that authors add temporal after monthly.

Response: We have added the word “temporal” to clarify the nature of the resolution.

 “The South American Amazon is one of the largest…” – should start as new paragraph since it diverts from the idea communicated in this paragraph.

Response: A new paragraph has been created at this location to ensure a more logical flow of ideas.

 “similar studies do not exist for the Amazon region” – Please confirm through a comprehensive literature search. I doubt this is entirely accurate.

Response: We have refined the text to clarify that while sub-regional studies exist, there is a scarcity of integrated, basin-wide, multivariate studies for the entire Amazon region.

 “this dataset encourages the creation of similar datasets” – please rephrase to improve clarity.

Response: We have rephrased this to emphasize that the dataset provides a framework/standard for the development of future longitudinal fire databases in other regions.

 “[-79.43629, -18.00816: -44.49108, 8.66346]” – Should not be in brackets.

Response: The brackets have been removed from the geographic coordinate strings.

 “MCD64A1” , “MCD12C1”, “MOD11C3” – please provide name of the product in addition to its ID or Acronymn.

Response: Full product names have been added alongside their respective IDs.

 “…acquired is (Integrated Multi-satellite Retrievals for GPM (Global Precipitation Measurement (GPM)…” – Should not be in brackets.

Response: The unnecessary nested parentheses have been removed for better readability.

 [89.75N–89.75S, 0.25E–359.75E] - Should not be in brackets.

Response: Brackets have been removed from the spatial extent definition.

 “…as –  -9999.” – not clear, please consider removing the dash.

Response: The dash has been removed to clarify that -9999 is the specific value used for missing data.

 “Noah 3.6.1, model…” – the comma here creates fragmentation.

Response: The comma has been removed to improve the sentence flow.

 “…and are termed Working Data” – The term has been used above, but only defined here. Please consider defining a term when they are first mentioned.

Response: The first use of *Working Data* has been corrected to ensure conceptual clarity. This and similar terms are introduced in the Introduction section, and defined and explained in the Data Processing section.

 “file (240 files over 20 years)” – please insert “i.e.,” in brackets.

Response: The text now has an added i.e., as recommended.

 There is space on the data link which breaks the hyperlink. Please correct.

https://doi.org/10.5281/ zenodo.7215402

Response: The space in the DOI URL has been removed.

# Reviewer report: Reviewer response for version 1

**DOI:** 10.5256/f1000research.181063.r418949

**Published:** 2025-10-27

**Authors:** I NENGAH SURATI JAYA

**Affiliations:** 1 IPB University, Bogor, Indonesia

Overall, this manuscript makes a meaningful contribution to advancing understanding of forest fire dynamics in the tropical Amazon through an integrative, data-driven approach.  This manuscript presents a comprehensive methodological framework for integrating heterogeneous spatial and temporal datasets to develop a high-resolution spatio-temporal database for forest fire analysis in the Amazon region. The approach is technically robust and highly relevant to large-scale environmental monitoring, forest management, and climate research.

 The novelty of the study lies not in producing new observations but in the harmonization of heterogeneous remote sensing and model-based datasets through rigorous preprocessing and geospatial standardization. This contributes a ready-to-use dataset that bridges gaps between data availability, accessibility, and analytical readiness—serving as a foundation for advanced environmental and fire research.

 While the study demonstrates methodological rigor and conceptual clarity, several areas require further elaboration to enhance the manuscript’s scientific depth, reproducibility, and practical impact. In particular, the data preprocessing procedures, validation strategy, and uncertainty assessment need to be described in greater detail to strengthen the transparency and reliability of the proposed workflow.

 Overall, this manuscript makes a meaningful contribution to advancing understanding of forest fire dynamics in the tropical Amazon through an integrative, data-driven approach.  This manuscript presents a comprehensive methodological framework for integrating heterogeneous spatial and temporal datasets to develop a high-resolution spatio-temporal database for forest fire analysis in the Amazon region. The approach is technically robust and highly relevant to large-scale environmental monitoring, forest management, and climate research.

 The novelty of the study lies not in producing new observations but in the harmonization of heterogeneous remote sensing and model-based datasets through rigorous preprocessing and geospatial standardization. This contributes a ready-to-use dataset that bridges gaps between data availability, accessibility, and analytical readiness—serving as a foundation for advanced environmental and fire research.

 While the study demonstrates methodological rigor and conceptual clarity, several areas require further elaboration to enhance the manuscript’s scientific depth, reproducibility, and practical impact. In particular, the data preprocessing procedures, validation strategy, and uncertainty assessment need to be described in greater detail to strengthen the transparency and reliability of the proposed workflow.

**Major Comments**

1. Data Integration and Resolution Consistency
  The integration of datasets with diverse spatial (250 m–37 km) and temporal (annual–monthly) resolutions may lead to interoperability inconsistencies. The authors should explicitly describe the *re-sampling* and *disaggregation* procedures, including interpolation methods and error assessments, to minimize spatial inaccuracy, particularly in environmentally heterogeneous regions.
2. Uncertainty of Model-Derived Variables
  The inclusion of model-based parameters (e.g., soil moisture, evapotranspiration) increases temporal completeness but introduces uncertainty. An *uncertainty analysis* and comparison with field or independent observational datasets are strongly recommended to enhance the reliability of the results.
3. Reprojection and Coordinate Transformation
  The reprojection of datasets to a standard coordinate reference system (e.g., EPSG:102033) must be carefully documented. The authors should describe the methods used to maintain geometric fidelity and correct for potential grid misalignments or boundary distortions.
4. Temporal Harmonization and Analytical Sensitivity
  Given the integration of variables with differing temporal frequencies (e.g., annual land cover versus monthly climate data), a clear *temporal harmonization framework* is needed. This should ensure that the resulting datasets retain temporal sensitivity for dynamic analyses such as fire risk modeling or temporal trend assessment.
5. Workflow Transparency and Quality Control
  With more than 240 temporal layers per attribute, reproducibility is a primary concern. The authors are encouraged to implement and document standardized *quality control procedures*—including metadata compliance (e.g., ISO 19115), workflow logs, and version tracking—to ensure process transparency and minimize accumulated processing errors.
6. Validation and Methodological Innovation
  Although the study effectively tackles dataset fragmentation and incompatibility using open-source solutions, its primary innovation lies in workflow standardization rather than algorithmic advancement. Incorporating validation experiments or developing a predictive model would strengthen the methodological contribution and scientific novelty of the work.

Are sufficient details of methods and materials provided to allow replication by others?

Partly

Is the rationale for creating the dataset(s) clearly described?

Yes

Are the datasets clearly presented in a useable and accessible format?

Yes

Are the protocols appropriate and is the work technically sound?

Yes

Reviewer Expertise:

Applied remote sensing and quantitative approach (machine learning/deep learning) in forestry and environment, e.g., forest fire, landslide, spatial modelling.

I confirm that I have read this submission and believe that I have an appropriate level of expertise to confirm that it is of an acceptable scientific standard, however I have significant reservations, as outlined above.

# Response

**Published:** 2026-01-18

**Authors:** Paula Moraga

**Affiliations:** King Abdullah University of Science and Technology Computer Electrical and Mathematical Science and Engineering Division, Thuwal, Makkah Province, Saudi Arabia

Thank you for your thorough review and insightful comments. We address each comment separately below.

 1. Data Integration and Resolution Consistency

 The integration of datasets with diverse spatial (250 m–37 km) and temporal (annual–monthly) resolutions may lead to interoperability inconsistencies. The authors should explicitly describe the re-sampling and disaggregation procedures, including interpolation methods and error assessments, to minimize spatial inaccuracy, particularly in environmentally heterogeneous regions.

Response: We have detailed our two-step alignment procedure in the Data Processing section (paragraphs 6 and 7). This includes an initial disaggregation of coarse datasets followed by a strategic resampling using the terra package in R. To ensure spatial accuracy across heterogeneous landscapes, we implemented "nearest neighbor" resampling for categorical/discrete variables to preserve data integrity, and "bilinear" interpolation for continuous atmospheric variables to accurately represent spatial gradients.

 2. Uncertainty of Model-Derived Variables

 The inclusion of model-based parameters (e.g., soil moisture, evapotranspiration) increases temporal completeness but introduces uncertainty. An uncertainty analysis and comparison with field or independent observational datasets are strongly recommended to enhance the reliability of the results.

Response: We agree that model-derived products involve inherent uncertainties. We have added a clarifying statement at the end of the Data Collection section explicitly defining the intended use of these parameters. While independent field validation is a valuable endeavor, it remains beyond the scope of this data curation manuscript. Our primary objective is to provide a standardized, analysis-ready infrastructure for the research community. The utility and reliability of this curated framework have already been demonstrated in a published application (Abid et al., 2025, Environ Ecol Stat, https://doi.org/10.1007/s10651-025-00661-x).

 3. Reprojection and Coordinate Transformation

 The reprojection of datasets to a standard coordinate reference system (e.g., EPSG:102033) must be carefully documented. The authors should describe the methods used to maintain geometric fidelity and correct for potential grid misalignments or boundary distortions.

Response: We have expanded the description of our coordinate transformation process in Data Processing (paragraph 4). The use of EPSG:102033 (South America Albers Equal Area Conic) is now explicitly justified as a measure to maintain geometric fidelity and minimize area/shape distortions across the basin’s longitudinal extent. To resolve grid misalignments, we describe the implementation of a standardized fixed spatial grid as a master template, ensuring identical geographical footprints for every pixel across all variables.

 4. Temporal Harmonization and Analytical Sensitivity

 Given the integration of variables with differing temporal frequencies (e.g., annual land cover versus monthly climate data), a clear temporal harmonization framework is needed. This should ensure that the resulting datasets retain temporal sensitivity for dynamic analyses such as fire risk modeling or temporal trend assessment.

Response: We have updated the Data Processing (paragraph 2) to define our "temporal expansion" framework, where annual/static variables are mapped consistently across corresponding monthly increments to create a synchronized 240-month time series. While we recognize that assessing the impact of data frequency on model sensitivity is an important research direction, such analytical performance testing is outside the scope of this work, which focuses on the workflow standardization and the resolution of dataset fragmentation.

 5. Workflow Transparency and Quality Control

 With more than 240 temporal layers per attribute, reproducibility is a primary concern. The authors are encouraged to implement and document standardized quality control procedures—including metadata compliance (e.g., ISO 19115), workflow logs, and version tracking—to ensure process transparency and minimize accumulated processing errors.

Response: We have updated the Data Processing (paragraph 8 and 10) and Technical Validation sections to highlight our quality control procedures. By utilizing a scripted algorithmic pipeline (terra package), we ensure that all 240 monthly layers are processed with identical parameters, effectively serving as an automated workflow log to eliminate manual intervention errors. While formal ISO-compliant certification is beyond the scope of this curation effort, our systematic documentation provides a transparent, reproducible framework already validated by its use in published dynamic research (Abid et al., 2025).

 6. Validation and Methodological Innovation

 Although the study effectively tackles dataset fragmentation and incompatibility using open-source solutions, its primary innovation lies in workflow standardization rather than algorithmic advancement. Incorporating validation experiments or developing a predictive model would strengthen the methodological contribution and scientific novelty of the work.

Response: We have added a clarifying statement in the Technical Validation section regarding the scientific novelty of this work. We believe that resolving dataset fragmentation through a standardized, analysis-ready framework is a critical scientific contribution that removes the primary bottleneck for modeling in the Amazon. While developing a new predictive model is beyond the current scope, the utility of this dataset has been proven in recent research (Abid et al., 2025), where the curated variables demonstrated the sensitivity required for complex ensemble modeling of fire dynamics.
